# Complement C3 deficiency alleviates alkylation-induced retinal degeneration in mice

**DOI:** 10.1186/s40662-022-00292-4

**Published:** 2022-06-09

**Authors:** Lu Du, Guang-Hua Peng

**Affiliations:** 1 Laboratory of Visual Cell Differentiation and Regulation, Basic Medical College, Zhengzhou University, 100 Science Ave, Zhengzhou, 450001 Henan China; 2 Department of Ophthalmology, Chinese PLA General Hospital, Beijing, 100039 China; 3 Department of Pathophysiology, Basic Medical College, Zhengzhou University, Zhengzhou, 450001 Henan China

**Keywords:** Complement C3, Retinal degeneration, DNA damage, Apoptosis, Photoreceptor cell

## Abstract

**Background:**

It has been found that the extensive use of anticancer drugs containing DNA-alkylating agents not only target cancer cells but also cause retinal inflammation through toxic intermediates. Complement C3 (C3) is a core component of the complement activation pathway, and dysregulation of the complement pathway is involved in several retinal degenerative diseases. However, whether C3 plays a critical role in alkylation-induced retinal degeneration is unclear.

**Methods:**

Following treatment with the alkylating agent methyl methane sulfonate (MMS), the C3 mRNA and protein level was measured, DNA damage and photoreceptor cell death were assessed in both wild-type (WT) C57BL/6J and C3 knockout (KO) mice.

**Results:**

We determined that complement pathway is activated following MMS treatment, and C3 knockout (KO) increased the rate of photoreceptor cell survival and preserved visual function. The mRNA levels of nuclear erythroid-related factor 2 (Nrf2) and related genes were higher after MMS application in C3 KO mice.

**Conclusion:**

In summary, our study found that C3 KO promotes photoreceptor cell survival and activates the Nrf2 signaling pathway in the context of alkylation-induced retinal degeneration.

**Supplementary Information:**

The online version contains supplementary material available at 10.1186/s40662-022-00292-4.

## Background

Retinal degenerative diseases threaten people’s vision through the gradual degeneration of photoreceptor cells and supporting cells [1]. Age-related macular degeneration (AMD) and retinitis pigmentosa (RP) are two major diseases involving the degeneration of photoreceptor cells. Currently, retinal degeneration is incurable and affects the lives of millions of individuals around the world [2]. Aging, genetic mutations and smoking have been identified as risk factors for retinal degeneration [3]. Alkylating agents, which are used as frontline chemotherapeutic drugs for cancer treatment, lead to extensive DNA damage in the body, which might cause retinal degeneration [4]. However, the pathological mechanisms of retinal degeneration remain unclear [5]. Therefore, it is important to elucidate the pathological mechanism underlying retinal degenerative diseases to aid the development of novel therapies.

It is well documented that oxidative stress induced by the environment and frontline chemotherapeutic drugs lead to DNA damage and apoptosis of photoreceptor cells [6]. Antioxidants have been found to partially alleviate the progression of retinal degeneration in rd1 mice (an RP model) [6]. A previous study also reported that retinal photoreceptor cells express P2X purinoceptor 7 (P2X7R) and that the P2X7 antagonist brilliant blue G (BBG), an approved adjuvant used in ocular surgery, prevents photoreceptor cell damage [7]. It has also been proven that DNA damage repair is tightly linked to retinal degeneration [8]. DNA damage can trigger the base excision repair (BER) pathway for alkylated DNA bases, and DNA damage is often repaired through BER; however, in some cells, BER intermediates can cause retinal cell damage [8]. It has been demonstrated that treatment with the alkylating agent methyl nitrosourea (MNU) leads to visual function impairment in rodent models [9]. In addition, Samson et al. reported that the application of methyl methane sulfonate (MMS) results in photoreceptor cell death in an animal model by activating the alkyladenine DNA glycosylase (AAG)-dependent signaling pathway [10]. Some anticancer agents, such as bleomycin and temozolomide, are used to kill cancer cells but often induce DNA damage, leading to retinal degeneration [8, 11]. Recently, it was proven that inflammation is involved in alkylation-induced retinal degeneration [8]. However, the exact downstream mechanism of alkylation-induced retinal degeneration needs further investigation.

The complement system plays an important role in retinal development and homeostasis [12]. C3 is a core component of the complement activation pathway [13, 14], and C3 activation is essential for the initiation of retinal impairment [15, 16]. C3 plays a key role in retinal degeneration, including AMD [17]. Upregulated expression and deposition of C3 have been observed in the degenerated retinas of both humans and animals [18–21]. Recent studies have found that inhibiting the complement activation pathway is a potential therapeutic strategy for AMD [22, 23]. However, whether C3 activation is involved in DNA-alkylating agent-induced retinal degeneration is unclear. Here, we found that activation of C3 caused extensive DNA damage and that MMS treatment led to photoreceptor cell apoptosis. Furthermore, knockout (KO) of C3 led to neuroprotection by increasing nuclear erythroid-related factor 2 (Nrf2) activity.

## Methods

### Animals

Eight-week-old C57BL/6J and C3 KO mice were used for this study. It was found that alkylation-induced retinal degeneration is sex dependent and that male animals are more sensitive to MMS [8, 24], so we chose to work with male mice for our study. C3 KO mice were kindly gifted by Professor Yusen Zhou (State Key Laboratory of Pathogen and Biosecurity, Beijing Institute of Microbiology and Epidemiology, Beijing 100071, China). All mice were housed in a temperature-controlled environment (21 °C ± 1 °C) on a normal 12-h light/dark cycle and provided ad libitum access to food and water. All animal protocols were conducted according to the ARVO Statement for the Use of Animals in Ophthalmic and Vision Research. The animal protocol was approved by the Institutional Animal Care and Use Committee of the General Hospital of Chinese People’s Liberation Army and the Academy of Military Medical Sciences (ID number: 307-ky-090).

### Treatment with MMS and tissue collection

MMS (Sigma, USA) was used to induce retinal degeneration. Male C57 and C3 KO mice were treated with a single i.p. injection of MMS at a sublethal dose of 75 mg/kg in saline [10]. After electroretinography (ERG), eyeballs were collected and fixed using 4% paraformaldehyde.

### Histology and immunofluorescence staining

After fixation for 24 h, the anterior segment of the eye was removed under a stereomicroscope, dehydrated by immersion in different concentrations of ethanol (70% ethanol for 20 min, 80% ethanol for 20 min, 90% ethanol for 20 min, 95% ethanol for 20 min, 100% ethanol for 20 min, and 100% ethanol for 20 min), cleared in xylene for 20 min twice and embedded in wax. Sections (5 μm) were prepared and used for hematoxylin and eosin (H&E) staining and immunofluorescent staining. The outer nuclear layer (ONL) thickness was evaluated throughout the whole retina [25]. After dewaxing, sections were subjected to antigen retrieval as previously described [26]. Primary antibodies were purchased from Abcam (anti-rhodopsin, ab221664, 1:600; anti-C3, ab225539, 1:600; mouse monoclonal anti-DNA/RNA Damage [15A3], ab62623, 1:600). All sections were stained under the same conditions. Negative control slides (no primary antibody) were used to set the confocal laser power and collection parameters. The same confocal parameters were used for all slides, and images of the retina at the same site were captured with a laser confocal microscope for all mice in each experiment. The fluorescence intensity in each image was measured with ImageJ [27]. Statistical analyses were performed using SPSS.

### ERG

Animals underwent ERG before and 1, 3, and 7 days after MMS injection as previously described [28]. Briefly, mice were dark-adapted for 12 h. Under dim red light conditions, the pupils were dilated with 0.5% tropicamide and 0.5% phenylephrine eye drop solution (Santen Pharmaceutical Co., Ltd., Osaka, Japan). The mice were anesthetized with isoflurane and kept warm to prevent hypothermia. A flashlight intensity of 0.5 log (cd s/m^2^) was used for all experiments. The ERG response was recorded with a corneal active gold electrode. The amplitudes of both the a and b waves were assayed.

### TUNEL staining

TdT-UTP nick end labeling (TUNEL) staining was performed according to the manufacturer’s instructions (Beyond, Shanghai). The slices were incubated with protease K (2 μg/mL) for 15 min at room temperature and washed three times with phosphate buffered saline (PBS) for 5 min each. Then, TUNEL solution was added for 30 min at 37 °C. After washing three times with PBS, DAPI was applied to counterstain the nuclei. Images were captured with a laser confocal microscope. ImageJ was used to process the images and semi-quantify the fluorescence intensity.

### Real-time qPCR (RT-qPCR)

The eyeballs were washed with clean cold PBS and then dissected on ice. The neural retina, retinal pigment epithelium (RPE) and choroidal tissue were quickly collected. mRNA was extracted with TRIzol (Thermo Fisher, USA), and cDNA was synthesized using iScript cDNA Synthesis kits (Bio-Rad, USA). RT-qPCR was performed as previously described [29]. Real-time PCR was performed using SYBR Green master mix (Bio-Rad, USA) on a CFX96 Bio-Rad system (Bio-Rad, USA) following the manufacturer’s instructions. The following PCR cycle parameters were used: 95 °C for 10 s and 40 cycles of 95 °C for 5 s and 60 °C for 34 s. Each reaction was performed in triplicates, and mβ-Actin1 was used as a reference gene. The primers used in this study are listed in Table 1.

**Table 1:** Primers list for real-time qPCR assay

Gene	Forward	Reverse
mC3	GAAGTACCTCATGTGGGGCC	CAGTTGGGACAACCATAAACC
mNrf2	GCCTTACTCTCCCAGTGAATAC	CCCAAATGGTGCCTAAGA
mSod2	CAGGATGCCGCTCCGTTAT	TGAGGTTTACACGACCGCTG
mNqo1	TGAAGAAGAAAGGATGGGAGG	AGGGGGAACTGGAATATCAC
mHmox-1	AGGTACACATCCAAGCCGAGA	CATCACCAGCTTAAAGCCTTCT
mRhodopsin	CCCTTCTCCAACGTCACAGG	GTAGAGCGTGAGGAAGTTGATG
mβ-Actin1	CGAGAAGATGACCCAGATCATGTT	CCTCGTAGATGGGCACAGTGT

### Statistical analysis

Statistical analysis was performed using SPSS 19.0 (Armonk, NY, USA). Two-tailed unpaired Student’s t test were used for statistical analysis. All quantitative values are presented as the mean ± standard error of the mean (SEM). Differences were considered significant at *P* < 0.05.

## Results

### The complement pathway is activated in the MMS-induced retinal degeneration model

**Fig. 1:**
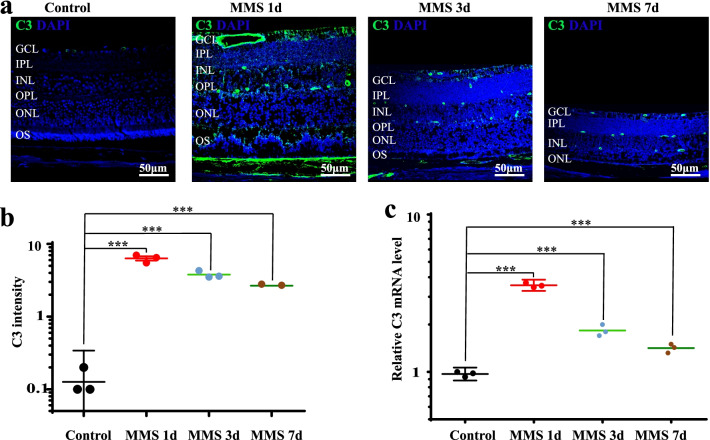
The complement system is activated in MMS-treated mouse retinas. **a** C3 expression in the retinas of MMS-treated mice was assessed by immunofluorescence staining before and 1, 3, and 7 days after MMS injection. **b** Semi-quantification of C3 immunoreactivity using ImageJ before and 1, 3, and 7 days after MMS injection. **c** C3 mRNA levels in MMS-treated mouse retinal tissue were evaluated by real-time qPCR and are expressed as the fold change compared with that in the control group after normalization to β-actin level (n = 6, ***P < 0.001 compared with control). MMS, methyl methane sulfonate; GCL, ganglion cell layer; IPL, inner plexiform layer; INL, inner nuclear layer; OPL, outer plexiform layer; ONL, outer nuclear layer; OS, outer segment

Previous studies have shown a strong connection between C3 and retinal degeneration [17]. However, the function of C3 in DNA-alkylating agent-induced retinal disease is unclear. MMS was administered to induce retinal injury [10]. Meira et al. observed profound loss of the photoreceptor layer after exposure to a medium dose of MMS (75 mg/kg) for 1 week. Therefore, we used 75 mg/kg MMS here. C3 protein was detected in retinal neural layers and the choroid layer (Fig. 1a). Semi-quantification of C3 expression revealed sharp upregulation of C3 expression 1 day after MMS injection. The expression level gradually decreased during degeneration but was still significantly higher in the MMS group than in the control group (Fig. 1a, b; *P* < 0.001, n = 6). We observed that the mRNA level of C3 was significantly higher in the MMS group than in the control group at 1, 3, and 7 days (Fig. 1c; *P* < 0.001, n = 6). These data indicated that upregulated expression of C3 may be correlated with the development of MMS-induced retinal degeneration.

### C3 deficiency preserves retinal function

**Fig. 2:**
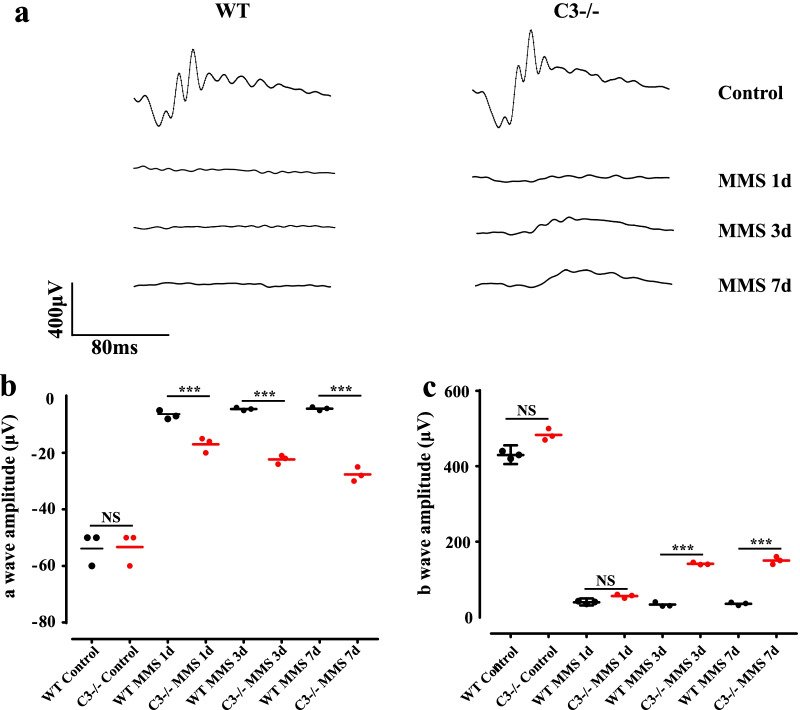
C3 knockout (KO) protects retinal function following MMS treatment. **a** Electroretinography (ERG) waves in wild-type (WT) and C3 KO mice before and 1, 3, and 7 days after MMS injection. **b**, **c** Dot plot of the amplitudes of a and b waves before and 1, 3, and 7 days after methyl methane sulfonate (MMS) injection (n = 6, ***P < 0.001 compared to the control)

Since we found an increase in the production of C3 in the MMS-treated mouse retina, we aimed to elucidate the function of C3 using the MMS-induced retinal degeneration model. We administered MMS (75 mg/kg) to wild-type (WT) and C3 KO mice and observed them for 1 week. MMS treatment led to an extinguished electroretinogram, with a sharp decline in the a- and b-wave amplitudes, in WT mice (Fig. 2a; *P* < 0.001, n = 6). On the other hand, we observed that retinal function was partially preserved in C3 KO mice, with a and b wave amplitudes being higher than those in littermate WT animals (Fig. 2b, c; *P* < 0.001, n = 6). This result suggested that genetic deletion of C3 had a remarkable effect on mitigating the damaging effect of MMS on retinal function.

### C3 deficiency protects against photoreceptor cell injury

**Fig. 3:**
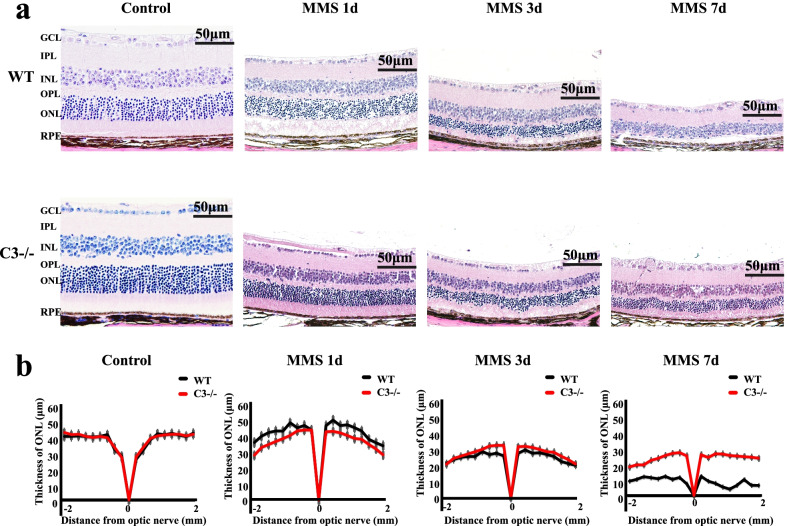
MMS retinal degeneration was alleviated in C3 knockout (KO) mice. **a** H&E staining of retinas from wild-type (WT) and C3 KO mice before and 1, 3, and 7 days after MMS injection. **b** Quantification of the ONL thickness in WT and C3 KO mice before and 1, 3, and 7 days after MMS injection (n = 6, ***P < 0.001 compared with control). MMS, methyl methane sulfonate; GCL, ganglion cell layer; IPL, inner plexiform layer; INL, inner nuclear layer; OPL, outer plexiform layer; ONL, outer nuclear layer; RPE, retinal pigment epithelium

**Fig. 4:**
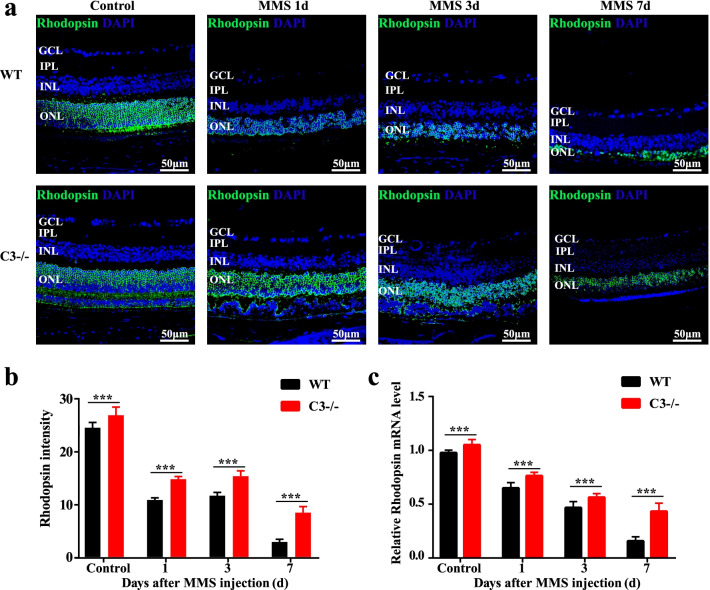
C3 knockout (KO) protects against a reduction in photoreceptor cell number. **a** Representative images for immunofluorescence staining of rhodopsin in retinal samples before and 1, 3, and 7 days after MMS injection. **b** Semi-quantification of the rhodopsin staining intensity in each group using ImageJ. C3−/− KO showed significantly more rhodopsin-positive cells than wild-type (WT) mice. **c** The rhodopsin mRNA level in MMS-treated mouse retinal tissue was evaluated by real-time qPCR and is expressed as the fold change after normalization to mβ-actin level (n = 6, ***P < 0.001 compared with control). MMS, methyl methane sulfonate; GCL, ganglion cell layer; IPL, inner plexiform layer; INL, inner nuclear layer; ONL, outer nuclear layer

Consistent with the data presented above, H&E staining of the retina showed disorganization of the photoreceptor layer from 1 day after MMS injection. Degeneration of photoreceptor cells was observed, and the thickness of the ONL was decreased in WT mice (Fig. 3a); however, photoreceptor cell degeneration was partially rescued in the C3 KO group from 1 to 7 days after MMS injection (Fig. 3a, b). These results suggested that activation of C3 in WT mice induced photoreceptor cell injury (*P* < 0.001, n = 6). Additionally, RT-qPCR and immunofluorescence staining showed that rhodopsin mRNA and protein levels were dramatically decreased in WT mice, while the degree to which rhodopsin mRNA and protein levels were decreased was significantly alleviated in C3 KO mice (Fig. 4a–c; *P* < 0.001, n = 6). In summary, these data indicate that loss of C3 dramatically reduces MMS-induced photoreceptor cell loss.

### C3 deficiency rescues photoreceptor cell apoptosis

**Fig. 5:**
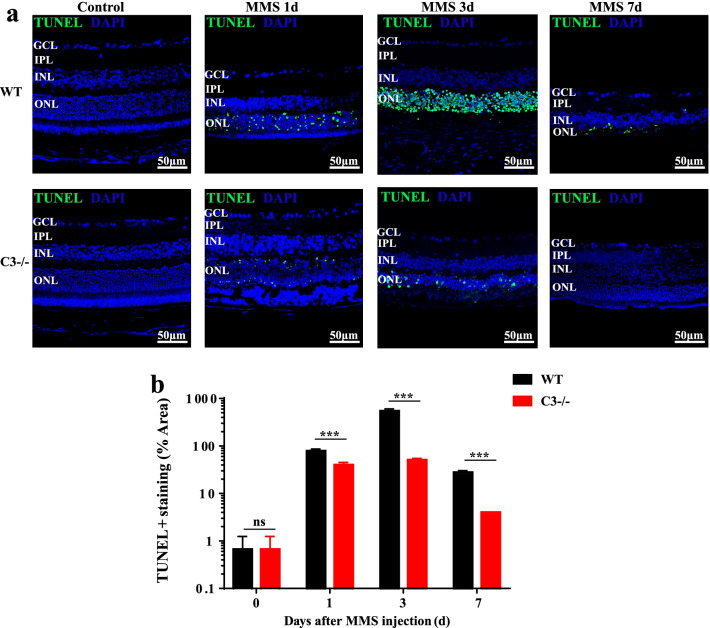
TUNEL staining of MMS-treated mouse retinas. **a** TUNEL staining before and 1, 3, and 7 days after MMS injection. **b** Semi-quantification of TUNEL staining in each group using ImageJ. There were fewer TUNEL-positive cells in the central retina in the C3 knockout (KO) mouse group than in the wild-type (WT) mouse group (n = 6, ***P < 0.001 compared with control). MMS, methyl methane sulfonate; GCL, ganglion cell layer; IPL, inner plexiform layer; INL, inner nuclear layer; ONL, outer nuclear layer

TUNEL staining was used to determine whether loss of C3 could rescue photoreceptor cell death. There were no TUNEL-positive cells before MMS treatment in either WT or C3 KO mice (Fig. 5a). As shown in the curve of the semi-quantification data, TUNEL-positive cells appeared in the ONL 1 day after MMS injection and the number of these cells reached a peak at 3 days and then gradually decreased in the WT group (Fig. 5b). The ratio of TUNEL-positive cells decreased in 3-day-old MMS-treated C3 KO mice compared with 3-day-old MMS-treated WT mice (Fig. 5a, b; *P* < 0.001, n = 6). Taken together, these showed that loss of C3 could effectively rescue photoreceptor cell apoptosis.

### C3 deficiency reduces retinal DNA damage

**Fig. 6:**
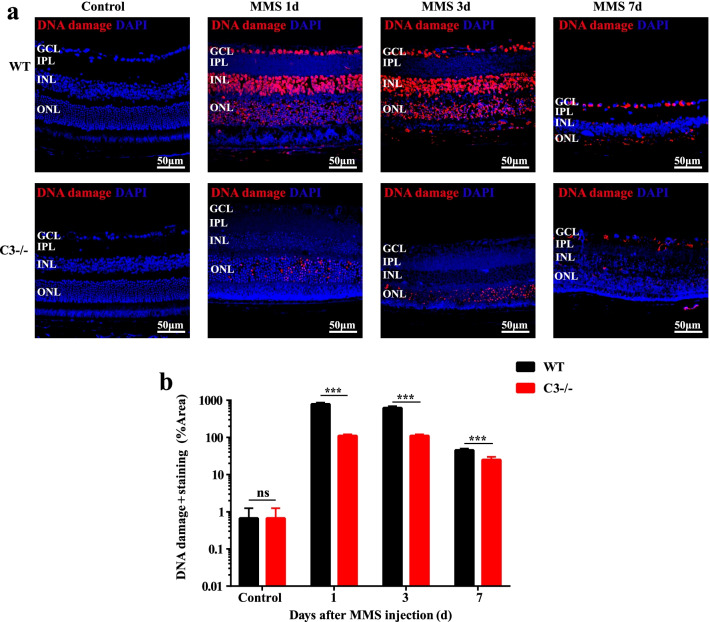
DNA damage in MMS-treated mouse retinas. **a** Representative images of DNA damage staining in retinal cross sections from each group before and 1, 3, and 7 days after MMS injection. **b** Semi-quantification of DNA damage staining in each group using ImageJ. The C3−/− mouse group significantly showed fewer DNA damage-positive cells in the retina than the wild-type (WT) mouse group (n = 6, ***P < 0.001 compared with control). MMS, methyl methane sulfonate; GCL, ganglion cell layer; IPL, inner plexiform layer; INL, inner nuclear layer; ONL, outer nuclear layer

To quantify and better characterize the mechanism of alkylation-induced retinal cell injury, the level of DNA damage was analyzed by immunofluorescence staining using an anti-DNA damage antibody. We detected extensive DNA damage in the positive group of NaIO_3_-induced oxidative stress retinal injury model (Additional file 1: Fig. S1). Extensive DNA damage was observed soon after MMS treatment in the WT group on 1 day, while the damage was limited to the ONL in C3 KO mice (Fig. 6a). Similar results were observed in 3- and 7-day samples (Fig. 6a). Moreover, the curve of the semi-quantified data showed that the ratio of DNA damage-positive cells decreased in the C3 KO group compared with the WT group (Fig. 6b; *P* < 0.001, n = 6). This result indicated that C3 deficiency reduced DNA damage not only in photoreceptor cells but also in whole retinal cells.

### C3 deficiency exhibits a Nrf2-mediated protective effect

**Fig. 7:**
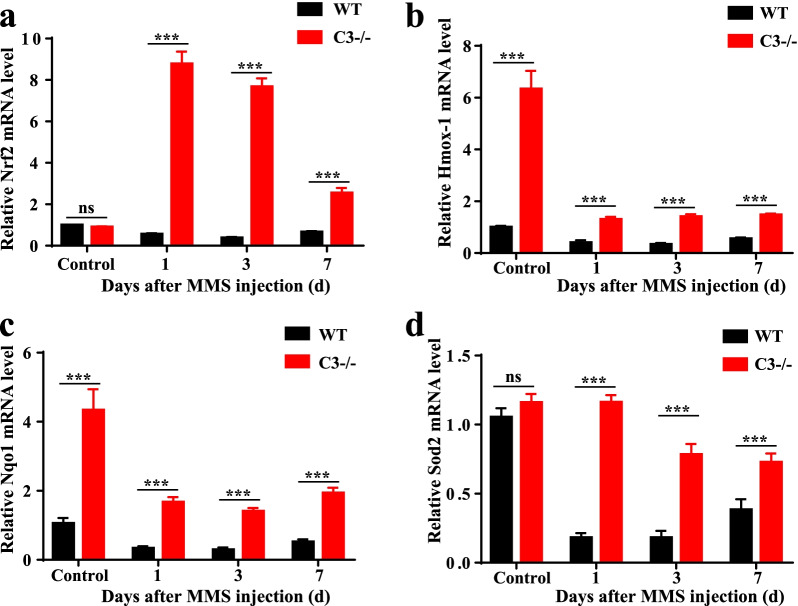
Oxidative stress level in MMS-treated mouse retina. **a**–**d** Nrf2, Nqo1, Hmox-1 and Sod2 mRNA levels in mouse retinal tissue were measured by real-time qPCR before and 1, 3, and 7 days after MMS injection and are expressed as the fold change compared with that in the control group after normalization to mβ-actin level (n = 6, ***P < 0.001 compared with control). MMS, methyl methane sulfonate; WT, wild-type

Furthermore, we determined whether C3 deficiency alleviates oxidative stress levels by measuring the mRNA levels of Nrf2, Nqo1, Hmox-1 and Sod2 before and 1, 3, and 7 days after MMS treatment. The mRNA levels of Hmox-1 and Nqo1 were upregulated in C3 KO mice compared with WT control mice. Nrf2, Nqo1, Hmox-1 and Sod2 mRNA levels were decreased in WT mice 1 day after MMS treatment (Fig. 7; *P* < 0.001, n = 6). The mRNA levels of these oxidative stress genes were upregulated in C3 KO mice compared to WT mice 1, 3, and 7 days after MMS treatment (Fig. 7; *P* < 0.001, n = 6). Taken together, these demonstrate that antioxidation was increased in C3 KO mice following MMS treatment and that C3 deficiency exerted neuroprotection via the Nrf2 signaling pathway.

## Discussion

C3 is a core immune system component that is essential for maintaining neural system homeostasis [30]. The function of C3 in alkylating agent-induced retinal degeneration in mice was studied in our experiments. C3 deficiency partially rescued photoreceptor cell apoptosis and preserved visual function by promoting the antioxidative signaling pathway and effectively attenuated a DNA alkylating agent-induced retinal degeneration.

Previous studies have found that AAG, a DNA repair protein, activates the BER pathway, which plays an important role in alkylation-induced retinal degeneration in animals [10]. AAG is an enzyme that can cleave the glycosyl bond connecting the base to the sugar phosphate backbone and then remove alkylated bases. This process generates a basic site that can be further processed by the BER machinery. Further research has revealed that the AAG-initiated base excision response is PARP1 dependent [24]. However, it is unclear whether inflammation is involved in alkylation-induced retinal degeneration. Allocca et al., however, demonstrated that inflammation played a crucial role in alkylation-induced retinal degeneration [8].

Further, we demonstrated that C3 activation contributes to photoreceptor cell apoptosis during alkylating agent-induced oxidative stress. This result is consistent with a previous report showing that inactivation of C3 alleviates retinal injury [31, 32]. In our experiments, we observed extensive DNA damage in retinal cells in wild-type animals, but only photoreceptor cells went through apoptosis. It seems that photoreceptor cells are more sensitive than other retinal cells to MMS. MMS treatment caused extensive DNA damage in retinal cells in wild-type mice. However, the damage to photoreceptor cells was limited in C3 KO mice. This was likely due to the protective effect of C3 deficiency. Moreover, we observed that C3 deficiency resulted in neuroprotection via Nrf2 activation. Activation of Nrf2 and downstream antioxidant-responsive elements (AREs) can protect neurons from damage and alleviate CNS disorders [33].

Nrf2 is an endogenous oxidative stress sensor. Under oxidative stimulation, Nrf2 can dissociate from Kelch-like ECH-associated protein 1 (Keap1) and activate the transcription of Sod2, Ho-1 and Nqo-1. Keap1 and Nrf2 play a central role in monitoring endogenous antioxidant enzyme activity. Here, MMS treatment significantly downregulated the expression of Nrf2, antioxidant enzymes, HO-1 and Nqo1. C3 deficiency partially reversed the downregulation of the expression of these genes. These results indicated that C3 could regulate the Nrf2-ARE pathway during retinal degeneration. However, C3 KO could not totally prevent retinal degeneration. Other inflammatory reactions might also be involved in alkylation-induced retinal degeneration. Additional work is therefore needed to elucidate the detailed mechanism underlying the effect of C3 on the Nrf2 signaling pathway.

## Conclusions

In summary, our study found that alkylating agent MMS treatment induced photoreceptor cell degeneration and activation of complement pathway. Moreover, C3 KO promotes photoreceptor cell survival and activates the Nrf2 signaling pathway in the context of alkylation-induced retinal degeneration. Our data suggests that inhibition of complement pathway might be helpful for treatment of alkylating agent induced retinal degeneration.

## Supplementary Information

**Additional file 1: Figure S1.** DNA damage in sodium iodate (NaIO_3_)-treated mouse retinas. a. Representative images of DNA damage staining in retinal cross sections from each group before and 7 days after NaIO3 injection. b. Semi-quantification of DNA damage using ImageJ. There are DNA damage-positive cells in the retina after NaIO_3_ injection (n = 6, ***P < 0.001 compared with control).

## Data Availability

Not applicable.
