# Supplementary figures and images for: Complement C3 deficiency alleviates alkylation-induced retinal degeneration in mice
Source: Eye Vis (Lond). 2022 Jun 9;9:22. doi: 10.1186/s40662-022-00292-4 (PMC9178834; doi:10.1186/s40662-022-00292-4)

**a**

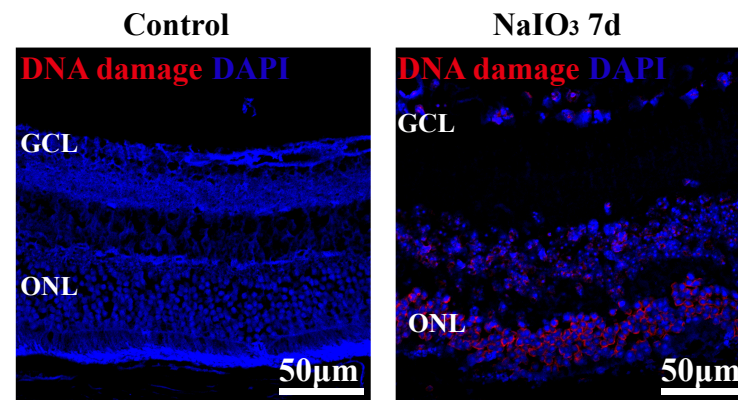

**b**

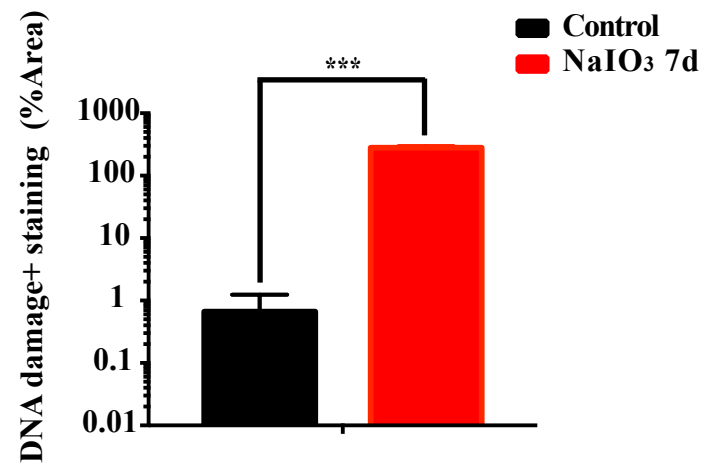

Supplement: Supplementary file 1 — Additional file 1: Figure S1. DNA damage in sodium iodate (NaIO3)-treated mouse retinas. a. Representative images of DNA damage staining in retinal cross sections from each group before and 7 days after NaIO3 injection. b. Semi-quantification of DNA damage using ImageJ. There are DNA damage-positive cells in the retina after NaIO3 injection (n = 6, ***P < 0.001 compared with control). [file 40662_2022_292_MOESM1_ESM.pdf]
